# Acute odontogenic infection combined with crowned dens syndrome: a case report

**DOI:** 10.1186/s13256-019-2084-x

**Published:** 2019-05-13

**Authors:** Tomoya Soma, Seiji Asoda, Moemi Kimura, Kanako Munakata, Hidetaka Miyashita, Taneaki Nakagawa, Hiromasa Kawana

**Affiliations:** Division of Oral and Maxillofacial Surgery, Department of Dentistry and Oral Surgery, Keio University School of Medicine, 35 Shinanomachi, Shinjuku-ku, Tokyo, 160-8582 Japan

**Keywords:** Pseudogout, Crowned dens syndrome, Odontogenic infection, Fever, Calcium pyrophosphate dihydrate crystal deposition disease, Case report

## Abstract

**Background:**

Calcium pyrophosphate dihydrate crystal deposition disease is a condition in which calcium pyrophosphate dihydrate crystal is deposited in joint cartilage and ligaments. Calcium pyrophosphate dihydrate crystal deposition disease that involves calcification around the odontoid process of the second cervical vertebra is called crowned dens syndrome. Crowned dens syndrome is accompanied by fever in addition to acute and intense neck, posterior head, and temporal pain; thus, distinguishing crowned dens syndrome may be difficult in the presence of odontogenic infection. To the best of our knowledge, this is the first report describing a patient with crowned dens syndrome with coexisting odontogenic infection.

**Case presentation:**

A 75-year-old Japanese woman was examined in the Emergency Department of this hospital due to a chief complaint of worsened buccal swelling on the left side. An odontogenic infection was considered, and she underwent her first examination. She presented with a body temperature of 37.4 °C, marked swelling and tenderness of her left lower eyelid through to her left cheek, and pain on the left temporal area. Blood tests revealed a leukocyte count of 6700/μL and a C-reactive protein level of 7.15 mg/dL. There was swelling and pain around the gingiva and acute purulent apical periodontitis of left maxillary second premolar. Cellulitis of the left cheek was diagnosed. After performing drainage of the pus, antibiotic treatment was initiated. Although her clinical symptoms improved, blood tests on day 9 of hospitalization revealed a leukocyte count of 6500/μL and a C-reactive protein level of 25.62 mg/dL, which were indicative of worsening symptoms. Computed tomography was performed to evaluate remote infection and images revealed a calcification around the odontoid process of her second cervical vertebra. When she was referred to the Orthopedic Surgery Department, pseudogout of the cervical spine was diagnosed. Subsequently, oral acetaminophen was initiated, and both her leukocyte count and C-reactive protein improved markedly.

**Conclusions:**

In the presence of persistent fever and abnormally high leukocyte and C-reactive protein indicative of an inflammatory reaction, coexistence of pseudogout should be considered. In particular, when symptoms of temporal pain are present, the possibility of pseudogout of the cervical spine must be considered in the differential diagnosis.

## Background

Calcium pyrophosphate dihydrate crystal deposition disease (CPPD) is a condition in which calcium pyrophosphate dihydrate crystal is deposited in joint cartilage and ligaments. Half of the cases of CPPD are asymptomatic; however, joint inflammation similar to gout, known as pseudogout, may occur. Contrary to gout, which is common in males of late middle age, pseudogout is more common in elderly females. Frequent sites of onset are the knees, hands, shoulders, feet, and elbow joints. Pseudogout may also occur in the cervical spine to lumbar spine [1–3], temporomandibular joint [4], and other locations; it may present with a variety of symptoms. CPPD, involving calcification around the odontoid process of the second cervical vertebra, is also known as pseudogout of the neck or crowned dens syndrome (CDS). As CDS is accompanied by fever in addition to acute and intense neck, posterior head, and temporal pain, distinguishing CDS may be difficult when odontogenic infection coexists. We present a case of CDS with coexisting acute odontogenic infection.

## Case presentation

Our patient was a 75-year-old Japanese woman. Two days prior to being examined at our hospital in January 2017, she became aware of mild bleeding due to gum injury caused by a maxillary denture fracture. After the initial bleeding, she experienced pain around the gingiva of the left maxillary second premolar (tooth No. 25) and left facial swelling, for which she was examined at the Emergency Department of our hospital. Acute odontogenic infection was considered, and she was referred to our department.

She had a history of aplastic anemia, renal dysfunction, bronchial asthma, and osteoporosis. She had no history of allergy, and she was currently taking orally administered cyclosporine, febuxostat, montelukast sodium, and theophylline. Epoetin beta pegol, a continuous erythropoietin receptor activator (CERA), was administered intravenously once a month.

**Fig. 1:**
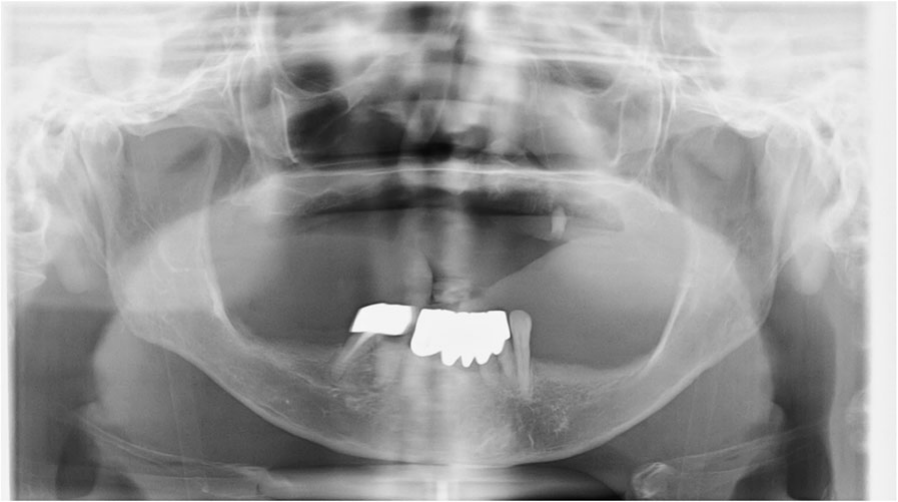
Panoramic radiograph. A transparent image is seen in the apical region of tooth No. 25

**Fig. 2:**
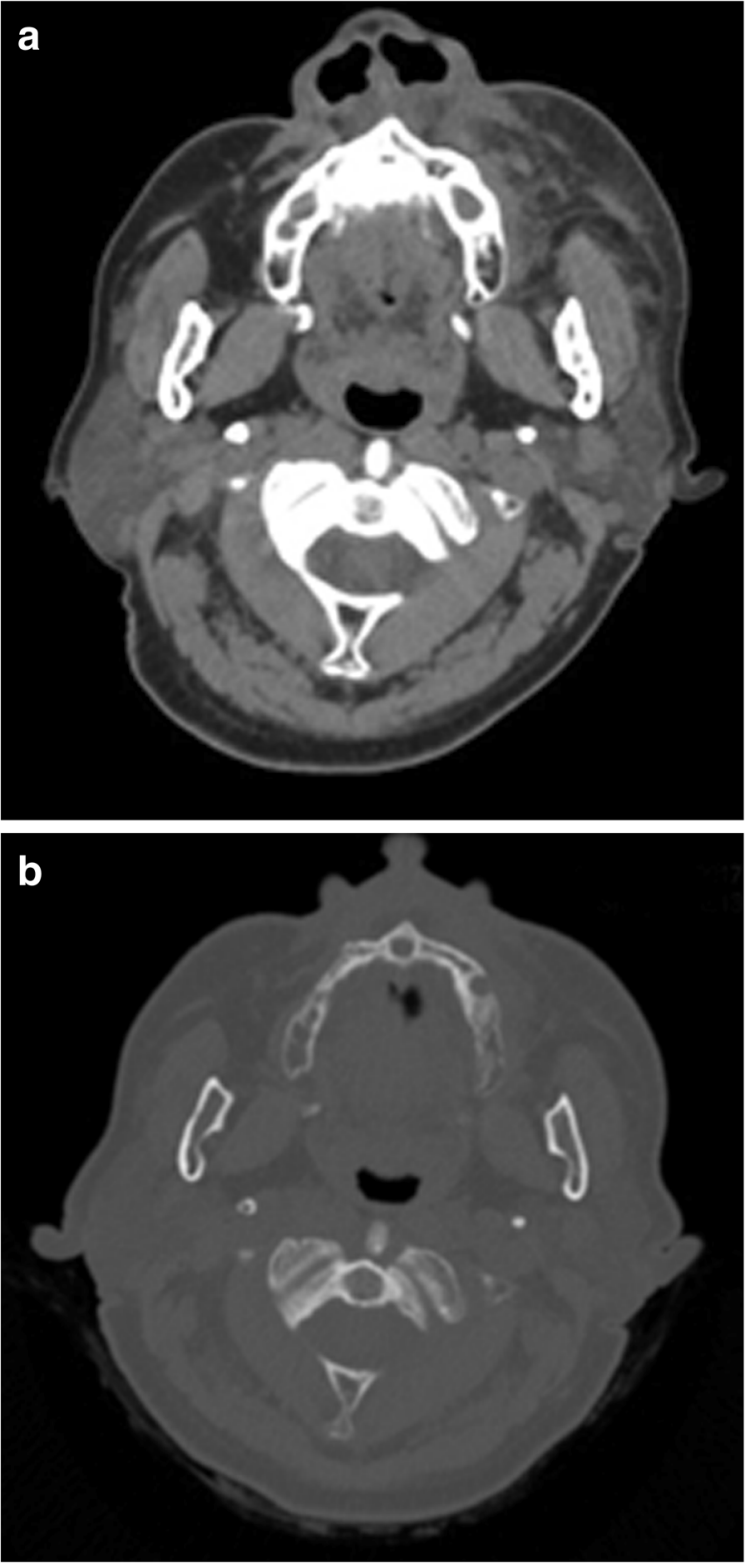
Simple computed tomography image of the neck at the first examination. **a** Axial image in soft tissue windows (width = 250, center = 30); widespread inflammation with adipose tissue opacities in the left cheek. **b** Axial image in bone windows (width = 2000, center = 400); a transparent image is seen in the apical region of tooth No. 25 in the left maxilla

On initial examination, she presented with marked swelling and tenderness from her left lower eyelid to her left cheek and to the left side of her upper lip. Spontaneous pain was present on the left temporal area. The stump of tooth No. 25 was intact, with swelling and tenderness around the gingiva. Pulsation was perceived upon palpating the buccal gingiva. A panoramic radiograph revealed radiolucency surrounding apical region of tooth No. 25. (Fig. 1). A simple computed tomography (CT) image revealed extensive inflammation, with adipose tissue opacities in the soft tissues of her left cheek (Fig. 2a). Although no abnormal image was seen in either of the maxillary sinuses, there was radiolucency surrounding apical region of tooth No. 25 (Fig. 2b). Blood tests at the initial examination revealed a leukocyte count of 6700/μL, which was a normal value; however, her C-reactive protein (CRP) concentration was high at 7.15 mg/dL. Our patient’s platelet count was slightly lower at 97,000/μL; this was attributed to her history of aplastic anemia. The blood urea nitrogen concentration was 34.1 mg/dL; the serum creatinine concentration was 1.43 mg/dL, which is indicative of renal dysfunction (Table 1).

**Table 1:** Laboratory data at initial examination

Test	Result	Normal range
WBC	6700/μL	(3500–8500/μL)
RBC	212 × 10^4^/μL	(370–490 × 10^4^/μL)
Hemoglobin	7.8 g/dL	(11.5–15 g/dL)
Hematocrit	23.50%	(35–45%)
Platelet	9.7 × 10^3^/μL	(150–350 × 10^3^/μL)
TP	6.8 g/dL	(6.7–8.2 g/dL)
ALB	3.9 g/dL	(3.9–5.2 g/dL)
T-Bil	1.0 mg/dL	(0.4–1.3 mg/dL)
AST	16 U/L	(10–35 U/L)
ALT	8 U/L	(5–40 U/L)
ALP	382 U/L	(100–320 U/L)
LDH	246 U/L	(5–40 U/L)
BUN	34.1 mg/dL	(8–20 mg/dL)
Cre	1.43 mg/dL	(0.4–0.8 mg/dL)
UA	6.5 mg/dL	(3.0–7.0 mg/dL)
Na	142.3 mEq/L	(136–145 mEq/L)
Cl	108 mEq/L	(99–107 mEq/L)
K	4.5 mEq/L	(3.6–4.8 mEq/L)
CRP	7.15 mg/dL	(0–0.35 mg/dL)

*ALB* albumin, *ALP* alkaline phosphatase, *ALT* alanine aminotransferase, *AST* aspartate aminotransferase, *BUN* blood urea nitrogen, *Cre* creatinine, *CRP* C-reactive protein, *LDH* lactate dehydrogenase, *RBC* red blood cells, *T-Bil* total bilirubin, *TP* total protein, *UA urea nitrogen*, *WBC* white blood cells. Inflammation coexisted with odontogenic infection. Although high C-reactive protein values were observed, the platelet count was slightly low, which was attributed to the patient’s history of aplastic anemia. Renal dysfunction was also observed

**Fig. 3:**
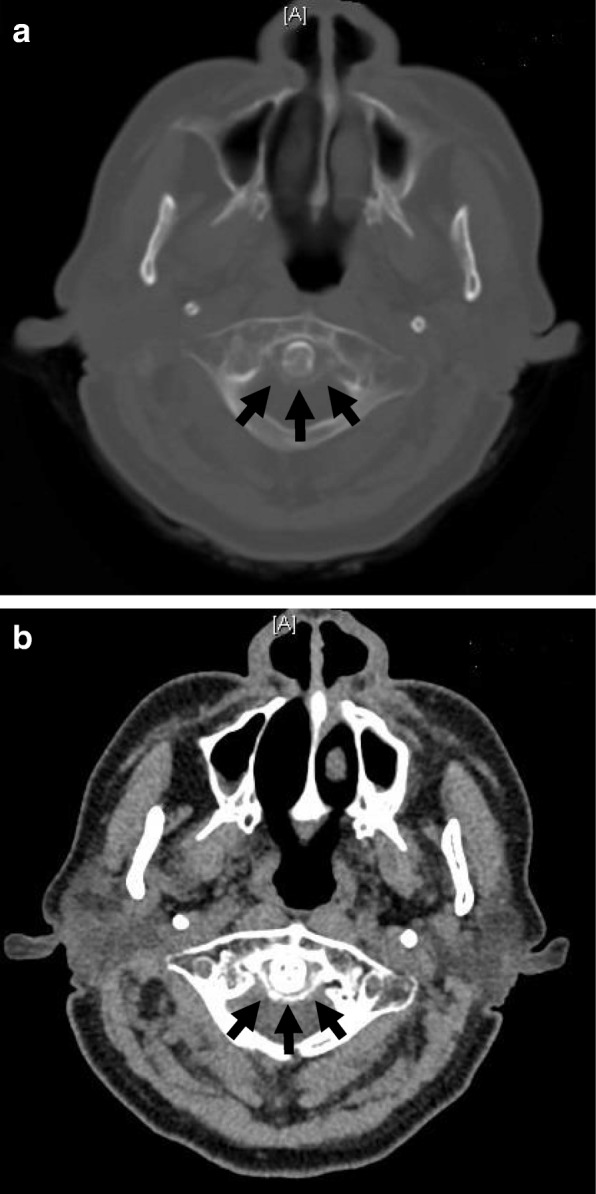
Contrast computed tomography image of the neck on day 10 of hospitalization. **a** Axial image in bone windows (width = 2000, center = 400); although there is evidence of calcification around the odontoid process of the second cervical vertebra, it is pale and fine (*arrows*). **b** Axial image in soft tissue windows (width = 250, center = 30); calcification around the odontoid process of the second cervical vertebra is clearly visible (*arrows*)

**Fig. 4:**
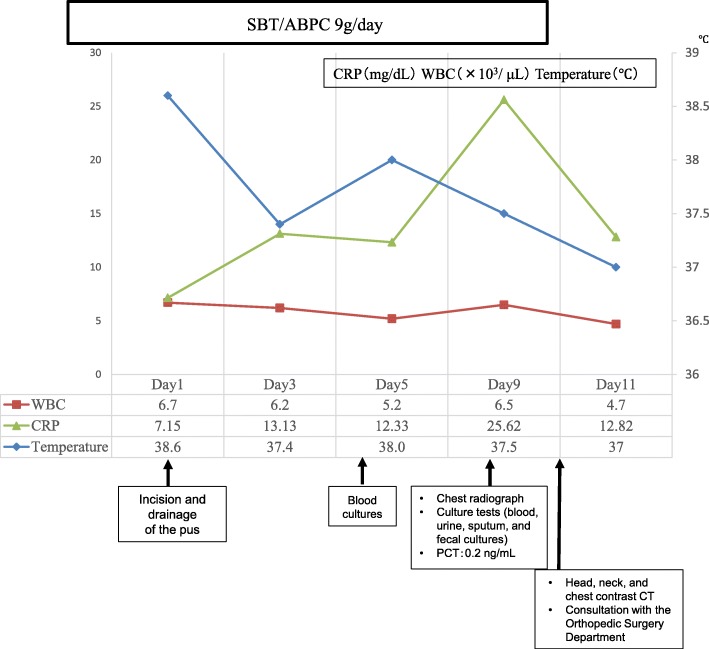
Progress after hospitalization. The patient was hospitalized immediately, and anti-inflammatory therapy with intravenously administered antibacterial agents was initiated. Computed tomography images were obtained on day 10 of hospitalization, calcification around the odontoid process of the second cervical vertebra was observed and crowned dens syndrome was diagnosed. *CRP* C-reactive protein, *CT* computed tomography, *PLT* platelets, *SBT/ABPC* ampicillin/sulbactam, *WBC* white blood cells

The buccal gingiva corresponding to the apical region of tooth No. 25 revealed swelling; as pulsation was perceived upon palpating the region, the Hematology Medicine Department of our institution was consulted regarding incision and drainage of the pus. Although anemia was present, her leukocyte count was normal, so it was considered safe to proceed with the intervention. On the same day, a fine-needle aspiration was performed under local anesthesia, and the suctioned pus was submitted for a bacterial culture test. Incision and drainage of the pus were then performed, followed by the placement of a gauze drain. She was hospitalized immediately, and anti-inflammatory therapy with intravenously administered ampicillin/sulbactam was initiated. As she had renal dysfunction, the dose of the antibiotic was lower than normal; ampicillin/sulbactam 9 g/day was administered. The Hematology Medicine Department was currently treating our patient with cyclosporine for aplastic anemia; after consultation, continuation of the oral treatment was permitted as long as her leukocyte count remained stable. Blood tests performed on day 5 of hospitalization revealed a leukocyte count of 5200/μL and a CRP value of 12.33 mg/dL, with no marked changes; however, she exhibited a fever of approximately 38 °C, so two sets of blood cultures were performed. Reddening around the gingiva of tooth No. 25, widespread facial swelling, and tenderness had all improved, but there was no improvement in the left temporal headache. In addition, there was onset of pain in the left side of her neck on the same day. Since the neck pain was mild, her condition was monitored by follow-up observations. Blood tests performed on day 9 of hospitalization showed a procalcitonin (PCT) concentration of 0.2 ng/mL, which was negative. Her leukocyte count was 6500/μL, and the CRP concentration was 25.62 mg/dL, indicating marked worsening of inflammation. A chest radiograph and culture tests (blood, urine, sputum, and fecal cultures) were performed to assess any remote infections, and the infection control team was asked to perform an examination. The chest radiograph revealed no evidence of pneumonia or other infections, and all culture tests were negative. In addition, inflammation of her left cheek improved, so the worsening of the inflammatory values could not be considered a causal factor. Head, neck, and chest contrast CT images were obtained on day 10 of hospitalization to more accurately identify the source of infection. Calcification around the odontoid process of the second cervical vertebra was observed and based on the history of temporal and neck pain, CDS was suspected (Fig. 3a, b). She was immediately referred to the Orthopedic Surgery Department for consultation, and CDS was considered the most likely cause of her increased leukocyte count and CRP value. Orally administered acetaminophen 600 mg/day was initiated to provide symptomatic therapy. Blood tests on day 11 of hospitalization revealed a leukocyte count of 4700/μL and a CRP level of 12.82 mg/dL, which constituted marked improvements. On day 13 of hospitalization, the symptoms were in remission, and she was discharged. Tooth No. 25, which was considered the cause of the buccal swelling on the left side, was extracted on a subsequent out-patient visit (Fig. 4). After tooth extraction, symptoms of neck pain and temporal pain improved, completing the orthopedic surgery intervention.

## Discussion and conclusions

CDS was first reported by Bouvet *et al.* in 1985 [1] and occurs predominantly in the elderly, with those aged ≥ 70 years accounting for at least 65% of cases. The male:female ratio is 3:5; thus, most patients with CDS are elderly women. Symptoms include acute and intense neck, occipital and temporal pain, as well as fever and other symptoms. Cervical CT images reveal characteristic calcification around the odontoid process of the second cervical vertebra, and CRP values are markedly higher than normal [5].

In pseudogout, crystals from the calcium pyrophosphate dihydrate crystal mass that flow into the surrounding tissues or joint cavities are phagocytosed by polymorphonuclear leukocytes, which trigger a series of biological responses [6, 7] causing disease. Furthermore, serious medical diseases including myocardial infarction can induce pseudogout [7]. The case reported here demonstrates, therefore, that systemic factors such as infection, including dental infection, probably contribute to the onset of pseudogout [8]. Moreover, inflammation of the C1–2 joints in CDS probably causes pain in the posterior neck of the greater occipital nerve area and the temporal area. Our patient also presented with temporal pain. Treatments reported to be effective include nonsteroidal anti-inflammatory drugs, colchicine, and corticosteroids. Our patient had a history of bronchial asthma and was, therefore, receiving oral bronchodilators. Based on her current treatments, the treatment protocol determined after consultation with the Orthopedic Surgery Department was low-dose orally administered acetaminophen alone and follow-up observation.

While differential diagnoses in orthopedic surgery usually focus on diseases such as meningitis, polymyalgia rheumatica, giant cell arteritis, osteomyelitis, and spinal tumor, misdiagnoses are common [2, 9]. To the best of our knowledge, this is the first report of a patient with CDS with coexisting odontogenic infection. In this case, there was clear evidence of odontogenic infection at the first examination, and antibiotic treatment yielded clear improvement of the clinical symptoms. Conversely, blood tests revealed worsening of the inflammatory findings. In such cases, the diseases that must first be considered causal factors of the fever and raised CRP levels are remote infections, such as urinary tract infection, catheter-related bloodstream infections, and hospital-acquired pneumonia. Exclusion of these possibilities is important. In this case, the infection control team was consulted after chest radiograph and various culture tests had been performed. Remote infection, including meningitis, could be ruled out. In addition, PCT is useful for distinguishing noninfectious disease from bacterial infection [10]. Compared with CRP, PCT has a faster reaction time and a long half-life. Moreover, PCT shows no correlation with CRP. PCT is primarily useful in the diagnosis of bacterial infection; even when medications are being used that readily affect leukocyte function, such as adrenocorticosteroids and anticancer drugs, PCT levels still increase. PCT has also been reported to increase under noninfectious states such as serious trauma, surgery, burns, neuroendocrine tumors, and pancreatitis, so a comprehensive interpretation is necessary. In this case, when PCT was measured on day 9 of hospitalization, the value was 0.2 ng/mL, which was negative; this result helped distinguish the condition from worsening infection.

It was difficult to capture the calcification surrounding the odontoid process of the second cervical vertebra by simple radiograph and magnetic resonance imaging, but diagnosis by CT proved effective. The characteristic image finding of CDS is a coronal, horseshoe-shaped calcification around the odontoid process of the second cervical vertebra. However, as calcification is pale and fine, and can thus be difficult to identify under normal bone windows (Fig. 3a), investigators have suggested that evaluations should also be performed under soft tissue windows [11]. Even in this case, it was difficult to identify calcification with bone windows (Fig. 3b); CDS was diagnosed based on soft tissue windows.

When pseudogout, including CDS, is diagnosed, the sites of calcium pyrophosphate dihydrate crystal deposition must be confirmed by imaging; other diseases, particularly infection, must be ruled out. Even with infection of the maxillary region of the oral cavity, the coexistence of pseudogout should be considered when abnormal clinical findings such as fever and high CRP values are observed. When temporal pain is observed, the possibility of CDS should be considered.

## Abbreviations

CDS: Crowned dens syndrome
CPPD: Calcium pyrophosphate dihydrate crystal deposition disease
CRP: C-reactive protein
CT: Computed tomography
PCT: Procalcitonin
Tooth number 25: Left maxillary second premolar
